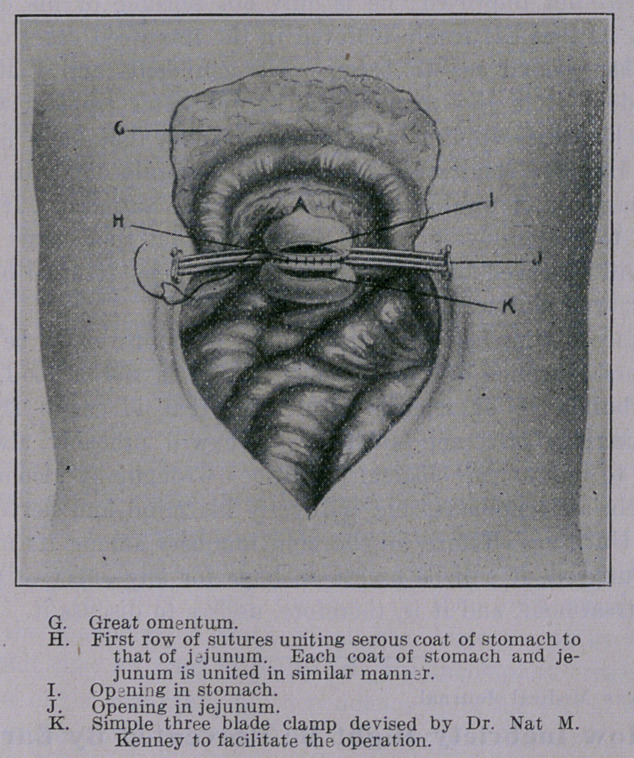# The Alcoholic Case and a Surgical Operation for the Cure of Chronic Alcoholism

**Published:** 1910-10

**Authors:** J. W. Kenney

**Affiliations:** San Antonio, Texas


### TEXAS MEDICAL JOURNAL.

Established July, 1885

F. E. DANIEL, M. D., - - - - - - Editor, Publisher and Proprietor

Published Monthly.—Subscription $1.00 a Year.

VOL. XXVI AUSTIN, OCTOBER, 1910. No. 4

The publisher is not responsible for the views of the contributors.

### Original Articles.

For Texas Medical Journal.

### The Alcoholic Case and a Surgical Operation For the Cure of Chronic Alcoholism.

J. W. KENNEY, M. D., SAN ANTONIO, TEXAS.

Several years ago the writer reached the conclusion that the profession of today was as far from a right summing up of the alcoholic case as were the surgeon-barbers of the fourteenth century. Many and varied articles have been written upon the subject dating from the time the North African ladies used alcohol as a dressing for the hair to the present time; but in the summing up—for the cure of the ravages wrought by its poison, ethyl, upon the human economy, not one step forward has been recorded. The best article that it was ever my privilege to read upon the subject concluded by recommending the whipping post as the best cure. Such a conclusion is simply a confession of ignorance, and an illustration of "man's inhumanity to man."

In the beginning of its career, alcohol was harmless, being chiefly used as a toilet article. Today it is the belly cheer of the world, and is disputing first place with the great white plague as a destroyer of the human race.

In this short paper it shall be my business to state scientific principles and to record observed phenomena; and in recommending a major surgical operation for the cure of chronic alcoholism I do so with the full knowledge of the criticism that will be measured out to me.

My reasons for resorting to the surgical treatment of chronic alcoholism were:

1. Because medical treatment fails in a majority, if not in all cases, and must necessarily continue to fail, because it does not remove the cause of the gnawing or craving for alcohol.

2. Because the pathological condition caused by alcohol is almost identical with pathological conditions produced by other causes which are relieved by surgical treatment.

The drunkard in giving a history of his case usually points to his stomach as the cause of his drinking. Very few claim that the taste for liquor has anything to do with it. In the stomach will also be found the first evidence that the physical basis of life —protoplasm—has been injured, and the physical structure of that organ is the first to present pathological conditions.

It has been proven that alcohol stimulates the action of the

Photograph of the abdomen of three cases taken four months after operation.

senses and organs of the body for a short time. This supra-normal condition is quickly followed by the infra-normal, which indicates a partial paralysis of the nerve ends and eventually of the nerve centers. Continued repetition of this process results in hardening and partial paralysis of the muscular coat of the stomach and an inflamed or ulcerated condition of the mucous coat. Such a condition produces a vitiated appetite and impairs the action of the stomach, which is the most important of the digestive processes. To relieve this condition, when produced by any agent other than alcohol, surgical measures are demanded. Why not apply the same principle in the alcoholic case?

Reasoning along this line convinced me that all hope of curing a case of chronic alcoholism lay in a surgical direction, and I resolved to try the operation that I had in mind upon the first case that would grant me the privilege. An ideal case soon presented itself—a young man of high degree, who had descended to the lowest depths of saloondom and had floundered about in it for several years, presented himself with the request that I do something for him. After explaining to him the operation that I proposed to do, he was removed to the sanatorium. After a two-weeks' preparatory treatment, a posterior gastro-jejunostomy by simple suture was performed upon him. He left the hospital thirty days later. This patient was about 35 years old; had been drinking for about ten years. During the last three years, business so interfered with his drinking that he quit business and drank day and night, consuming from one to two quarts of whisky

A. Transverse Colon turned up.
B. Posterior layer of great omentum.
C. Incision through posterior layer of great omentum.
D. Loop of jejunum.

during the twenty-four hours. He was one of the best known drunkards in the city, and no one could remember having seen him sober during the three years preceding this operation, and he was looked upon as past redemption and absolutely valueless, from a business standpoint. Today he is assistant manager of a large mercantile establishment in this city and a sober, respected citizen.

Case No. 2 is that of a young dentist about 30 years of age. He stated that for several years he had consumed all the liquor he could get during the day and took a bottle to bed with him at night. His constant drinking had made a veritable neurasthenic of him. He consulted me regarding the operation, and was advised to have it performed. He then consulted a number of medical friends, and was advised by each one of them not to have the operation performed, one or two going so far as to get down their anatomies and surgeries to demonstrate the absurdity of the thing. He recognized the sincerity of his friends' advice but finally came to the sanatorium and had the operation performed. From a vagabond dentist, hounded by dozens of people whose money he had taken during half-sober intervals as advance payments on work which he was never able to perform, he is now, and has been ever since the operation, a sober man, and no one hesitates to trust him with their work.

E. Pouch of stomach drawn through incision in posterior layer of omentum and out of abdomen with clamp applied.
F. Loop of jejunum selected at a point about 15 inches from duodenum and held in opposition to stomach by clamp.

Case No. 3 is that of a traveling man about 35 years of age. He had been drinking at irregular intervals for a number of years. The intervals had gradually grown closer together until life became just one long drunk. Being deserted by wife and family and given up as hopeless and discharged by his employer, he decided that something had to be done. Upon the advice of a former disciple of Bacchus, whom I had operated upon, he entered the sanatorium and had the same operation performed. He is today united with his family, holds a good position, and points with pride to the scar upon his abdomen as the doorway by which he escaped the drink demon.

These cases have been selected at random from a series of seventeen. The result has been equally as good in all the cases, with the exception of two—one dying and the other relapsing. The death took place two days after the operation, and was due to angina pectoris. The patient was a chronic sufferer from this ailment and other pains, in limbs, etc. The relapsing case was that of a middle-aged, half-witted man. He was evidently sent to me with the hope that the operation would kill him, for no sooner did I announce the fact that he had passed the danger point than his wife and brother-in-law wanted to take him home.

G. Great omentum.
H. First row of sutures uniting serous coat of stomach to that of jejunum. Each coat of stomach and jejunum is united in similar manner.
I. Opening in stomach.
J. Opening in jejunum.
K. Simple three blade clamp devised by Dr. Nat M. Kenney to facilitate the operation.

This they did over my protest, on the ninth or tenth day, and placed him in a filthy room a few feet from his wife's beer saloon, where former friends congregated and drank about his bed. The only reason that I can now give for having operated upon this man is that I possessed the zeal of a recent convert to the curative properties of surgery in chronic alcoholism. My zeal has, therefore, placed at the door of the operation this one failure; it will have to stand as an object lesson for the future.

The operation as performed by me in these cases is described in connection with the photographic illustrations presented herewith.

It is not my purposes to advocate so grave a surgical procedure in all cases of chronic alcoholism, but only in those apparently hopeless cases where everything else has failed, and the patient is still in fair mental and physical condition and wants to be cured. The result at my hands has thus far exceeded expectations. Dr. Johnson said of the orator under the stimulation of alcohol: "Before dinner, men meet with great inequality of understanding, and those who are conscious of their inferiority have the modesty not to talk. When they have drunk, every man feels happy, and loses that modesty and grows impudent and vociferous, but he is not improved; he is only not sensible of his defects." It may be that the result achieved in the seventeen cases operated upon has caused me to become over-confident, and that I am affected by it in like manner as Dr. Johnson's tipplers were affected by their tippling. However, I know that such is not a fact. Had the result of the operation been only one-half as good as it has been, I would have been more than satisfied. It is presented to the profession with the expectation that many drunkards will be cured by it and the world thereby relieved of much misery, woe and want.

In face of the fact that alcohol has been proven to be one of the worst enemies of protoplasm—that it is not a food, for it never builds up or repairs tissue, and that it passes from the body unassimilated and unchanged, we will probably always be forced to relieve pathological conditions wrought by alcohol upon the inebriate's stomach, and indirectly his mind and nervous system. Until each family in the land numbers among its members one drunkard, it will be useless to hope for any effective prophylactic treatment, and it is, therefore, uselses to discuss it.

## Figures and Tables

**Figure f1:**
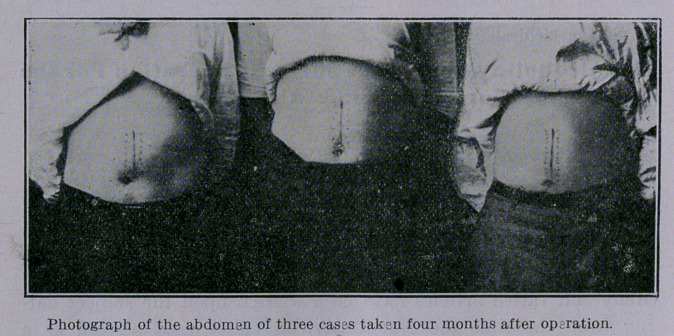


**Figure f2:**
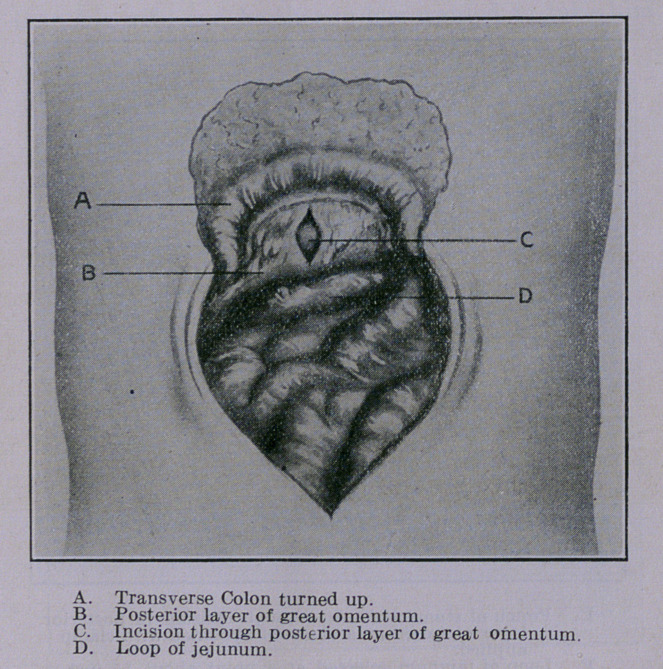


**Figure f3:**
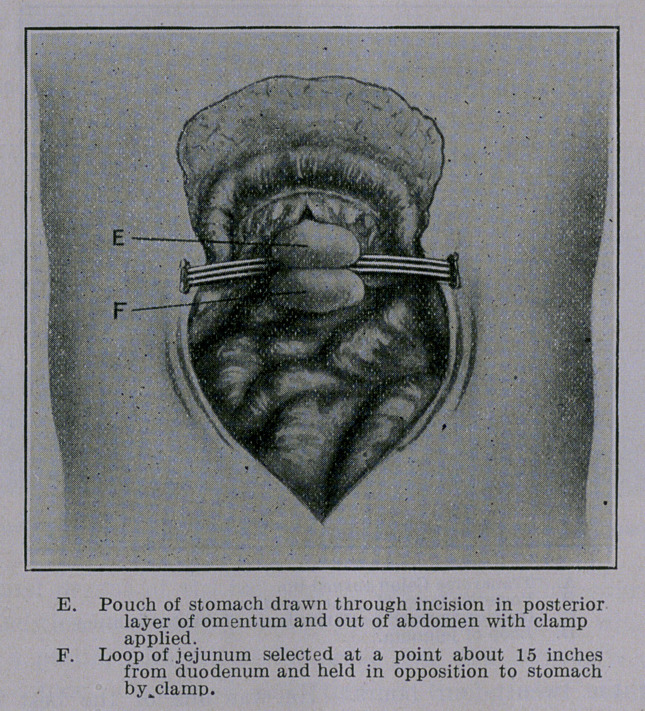


**Figure f4:**